# Synergistic effect of hydroxyapatite-titania nanocomposites derived from machining waste on the corrosion resistance of Ti6Al4V implants

**DOI:** 10.1038/s41598-026-47113-5

**Published:** 2026-05-04

**Authors:** Majid Kavanlouei, Mehrdad Shahbaz, Seyyed Amir Reza Alavizadeh, Amir Ahmadi Khoshalani

**Affiliations:** Department of Materials Science and Engineering, Faculty of Engineering, Urmia University, Urmia, 5756151818 Iran

**Keywords:** Electrophoretic deposition, Sol-Gel method, Corrosion, XRD, SEM, Chemistry, Engineering, Materials science, Nanoscience and technology

## Abstract

In this study, hydroxyapatite (HA) was synthesized via the sol–gel method and characterized to ensure phase purity and nanoparticle formation. Titanium machining chips were recycled and transformed into nanopowder using planetary ball milling to enhance material sustainability. Composite coatings of HA–TiO_2_ were subsequently deposited onto Ti6Al4V alloy substrates through electrophoretic deposition (EPD) at a constant voltage of 45 V for 90 s, with HA contents of 10, 20, 30, and 40 wt%. After drying at ambient conditions, the coated samples were vacuum-sintered at 1100 °C for 4 h. Microstructural and phase analyses conducted by SEM and XRD revealed the formation of dense, homogeneous coatings composed of Ti, HA, and oxide phases. Based on EIS and polarization analyses in simulated body fluid, the corrosion resistance of all coated samples was considerably superior to that of the untreated Ti6Al4V alloy. Among them, the coating containing 30 wt% HA demonstrated the highest corrosion resistance, which was attributed to its uniform and stable phase distribution. Overall, this study presents a cost-effective and sustainable strategy to recycle Ti6Al4V machining chips for developing biocompatible coatings with superior corrosion protection for metallic implant applications.

## Introduction

Titanium and its alloys are widely used for orthopedic and dental implants due to their favorable mechanical properties, corrosion resistance, and biocompatibility. However, increasing evidence indicates that their long-term performance in physiological environments is still challenged by localized corrosion, tribocorrosion, and the gradual degradation of the native oxide layer, particularly under cyclic loading and inflammatory conditions. These phenomena may lead to metal ion release and adverse biological responses, ultimately compromising implant longevity^1–3^.

During the industrial production of different types of implants, tons of machining chips are produced as waste materials^4^. Proper treatment of these wastes is essential to protect the environment and reduce disposal costs, while simultaneously enabling the development of cost-effective technologies for advanced materials^5^. Identifying a suitable method for recycling and reusing this waste is of significant economic, strategic, and environmental importance^6^. Various methods have been proposed to achieve this, including leaching and milling methods^7^. One of the advantages of the high-energy ball milling method is its industrialization and low cost^8^. Mechanical alloying is a versatile technique for synthesizing nanocomposites with tailored microstructures^9^. The recycling of titanium machining chips into raw materials aligns with circular economy principles, reducing waste and feedstock costs^10^. Solid-state recycling methods, such as mechanical alloying (MA), enable the conversion of metallic chips into high-quality powders^11–14^. For instance, Soufiani et al.^15^ successfully produced nanostructured Ti6Al4V/Al₂O₃ composites from residual scraps, while Teja et al.^16^ fabricated Ti–TiC composites using recycled chips. These studies highlight the viability of MA for repurposing titanium waste into advanced materials.

Recent comprehensive reviews and experimental studies have emphasized that surface chemistry, oxide stability, and interfacial reactions play a decisive role in governing both corrosion behavior and biological integration of Ti-based implants^17,18^. Recent publications have emphasized the critical role of surface engineering in tailoring the interfacial properties of titanium alloys, including oxide stability, corrosion behavior, and functional integration with ceramic or bioactive phases^19,20^.

Hydroxyapatite, a bioactive ceramic mimicking bone mineral composition, is extensively used to improve titanium’s osseointegration^21^. Surface coatings of nano-HA have demonstrated significant osteogenic potential^22^, while hybrid composites like HAp/TiO₂^23^ and graphene/ HAp synergize mechanical strength with bioactivity^24^. By combining titanium chips with bioactive additives like HA, nanostructured powders can be engineered to achieve homogeneous dispersion of reinforcing phases. Previous works on Ti–HA composites via PM routes underscore their potential for orthopedic applications, though challenges in achieving nanoscale homogeneity persist^25,26^. Furthermore, studies on HA-reinforced Ti6Al4V processed via laser powder bed fusion (LPBF) and selective laser melting emphasize the importance of particle size and distribution in optimizing mechanical and biological performance^27,28^.

While mechanical alloying (MA) has successfully converted Ti6Al4V chips into nanostructured powders, the direct application of these powders to create functional surfaces remains a challenge, often requiring energy-intensive subsequent processing steps. Conversely, electrophoretic deposition (EPD) offers a scalable and cost-effective route for depositing bioactive coatings like HA onto metallic substrates^29,30^. However, studies utilizing EPD often employ commercially sourced HA, neglecting the economic and environmental benefits of integrating in-situ synthesized, recycled additives^31^.

In contrast to these previous approaches, the present work introduces a direct, low-cost, and environmentally sustainable strategy utilizing real machining chips derived from industrial implant manufacturing. By applying a simplified mechanical milling technique, these waste Ti6Al4V chips are transformed into fine composite powders without resorting to expensive atomization or melting procedures. Subsequently, the recycled powders are combined with in-situ synthesized nano-HA and deposited via a cost-effective electrophoretic deposition (EPD) process to form a uniform and adherent HA–TiO_2_ coating. This dual-stage, economically optimized pathway distinguishes the current study from existing literature by directly coupling solid-state recycling with surface functionalization. The resultant coatings exhibit improved corrosion resistance and bioactivity, validating the feasibility of producing high-performance biomedical surfaces from industrial titanium waste. This work therefore presents a practical and financially viable route toward sustainable material development and enhanced implant performance through the synergistic combination of low-cost powder recycling and electrophoretic HA coating.

## Materials and methods

### Synthesize hydroxyapatite powder via a sol-gel method

**Fig. 1:**
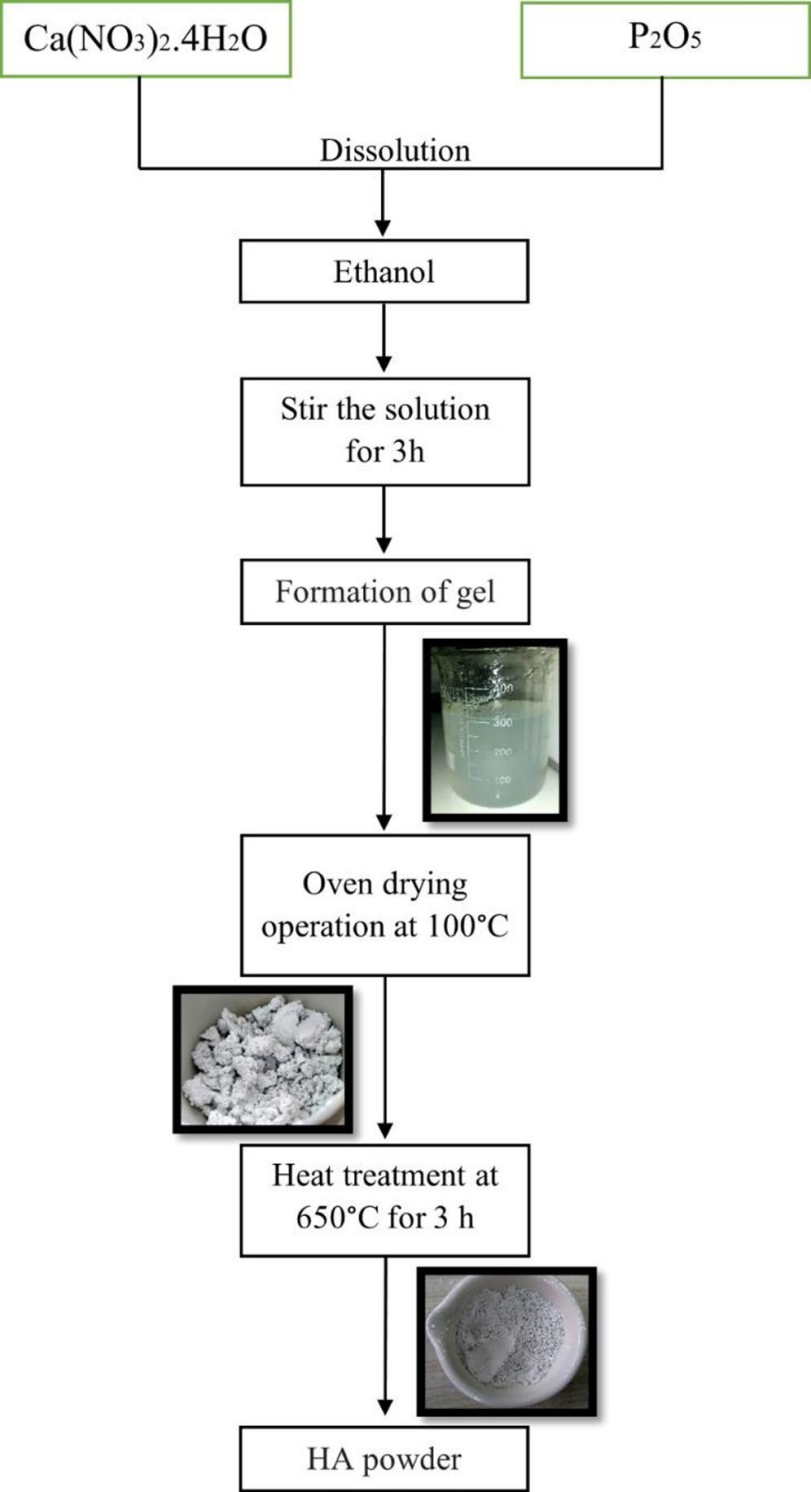
Schematic representation of the hydroxyapatite HA synthesis pathway utilizing the sol-gel method.

Hydroxyapatite (HA) powder was synthesized utilizing a sol-gel technique, employing calcium nitrate tetrahydrate (Ca(NO₃)₂·4 H₂O, Merck) and phosphorus pentoxide (P₂O₅, Merck) as the primary precursors. Stoichiometric Ca/P molar ratios of 1.67 were achieved by separately preparing 0.5 mol/L P₂O₅ solution and a 1.67 mol/L Ca(NO₃)₂·4 H₂O solutions in absolute ethanol. Continuous magnetic stirring (maintained for 2 h) was implemented during the controlled mixing of these solutions to inhibit rapid gelation, thereby facilitating homogeneous gel network formation. The resulting gel was subsequently dehydrated in an electric oven at 100℃ for 20 h, followed by calcination at 650 ℃ for 3 h under ambient atmospheric conditions to ensure the crystallization of the desired HA phase. The schematic illustration of the sol-gel synthesis approach employed for HA is presented in Fig. 1.

### Milling of Ti6Al4V chips to produce nanopowders

Ti6Al4V machining chips, sourced from medical implant manufacturing, were cleaned via sequential acetone immersion and ultrasonic bath treatment to remove organic contaminants and machining oils. The spiral-shaped chips were mechanically crushed using a mortar and pestle to produce coarse particles, which were further refined through planetary ball milling (Amin Asia Instrument Plant). The milling chamber, composed of high-chromium hardened steel, was loaded with a 50:50 ratio of 14 mm and 10 mm carbon steel balls. Milling was conducted at 500 rpm under ambient conditions, with intermittent 30-minute pauses to prevent overheating. A process control agent (PCA) was introduced at 1.5 wt% to mitigate particle agglomeration during milling.

After 5 h of preliminary Ti6Al4V milling, the synthesized HA powder was incorporated into the suspension at 10, 20, 30, and 40 wt% ratios.

### Electrophoretic deposition

For suspension preparation, 0.6 g.L iodine was added to ethanol (i.e. C_i_=0.6 g/L) and magnetically stirred for 30 min; then proper amounts of milled particles; leading to C = 5 g/L, was added into the suspension and magnetically stirred again for 30 min. Finally, various amounts of HA (i.e. C_Al_=0, 10, 20, 30 and 40 wt%) were added to the suspension and magnetically stirred for 1 h. Furthermore, before deposition, the suspensions were subjected to agitated by ultrasonic waves produced using a Hielscher-UP100H probe ultrasonicator (Ultrasound Technology, Germany) for 30 min.

**Fig. 2:**
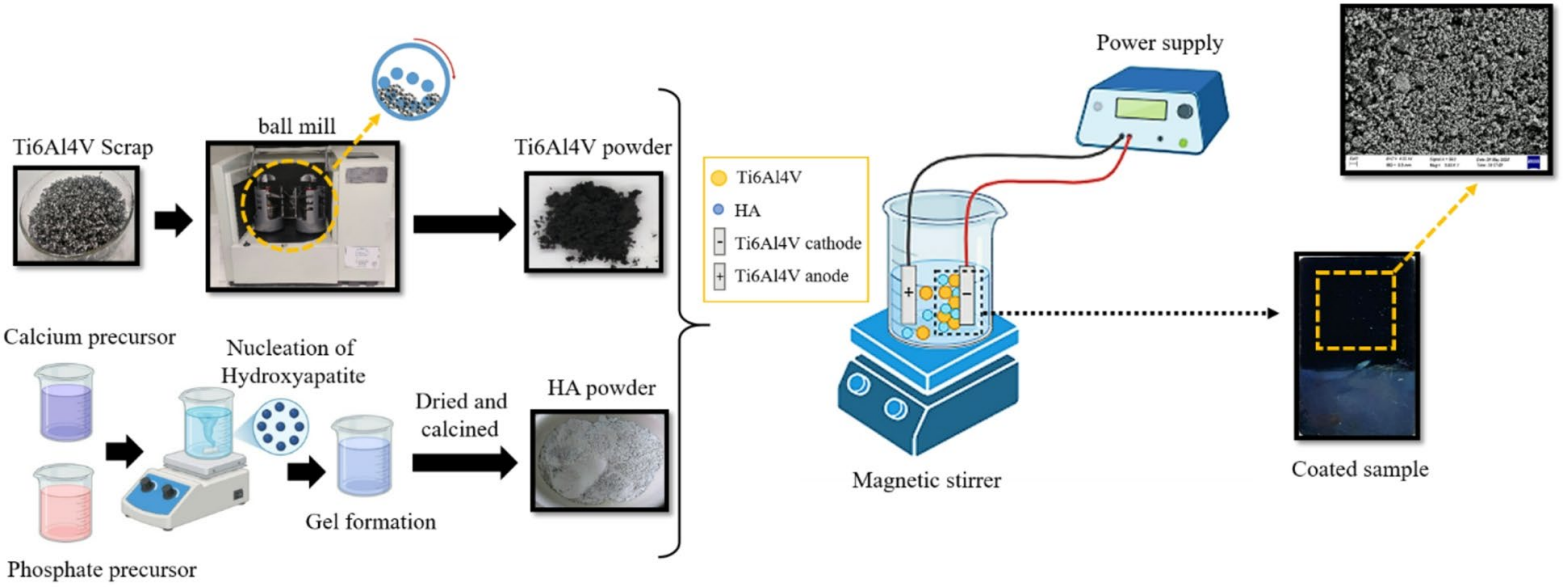
Comparative schematic illustrations of the synthesis stages: planetary ball milling, sol-gel preparation, and the final electrophoretic deposition (EPD) process.

Electrophoretic deposition (EPD) was executed within a two-electrode cell setup. Titanium alloy substrates (Ti6Al4V plates, dimensions 15 × 10 × 2 mm^3^) served as the working electrode, positioned parallel to a Ti6Al4V plate counter electrode of comparable size. The electrodes were separated by a fixed working distance of 10 mm within the deposition cell. Prior to coating, the substrates, obtained via wire cutting from a thick (2 mm) sheet, underwent rigorous surface preparation. This involved initial mechanical polishing using abrasive papers ranging from P80 to P1200 (FEPA grading). Subsequently, the surfaces were subjected to SiO_2_​ particle blasting at a pressure of 0.5 MPa 5 bar) for 30 s. Final cleaning involved sequential ultrasonication for 15 min in both acetone and ethanol. The coatings were subsequently deposited by applying a constant voltage of 45 V for a deposition time of 90 s, managed by a regulated DC power supply (Mastech, Model HY30001E, USA). The schematic of the process use for produce nanopowders from chips and subsequent EPD process is shown in Fig. 2.

### Characterization

Phase composition and crystallographic evolution were analyzed via X-ray diffraction (XRD; PHILIPS-PW1800, CuKα, λ = 1.5406 Å) over a 2θ range of 10°–90° (step size: 0.05°, step time: 0.5 s). Morphological and elemental analyses were conducted using scanning electron microscopy (SEM; Leica Cambridge Stereoscan S360) coupled with energy-dispersive X-ray spectroscopy (EDAX). To account for surface irregularities and inhomogeneous powder distribution, EDAX mapping was performed across multiple regions, and oxygen content was cross-validated with XRD data. Particle size distribution was statistically evaluated using Clemex image analysis software (Clemex Technologies Inc, Canada, https://www.clemex.com)^32^. Functional group identification in HA was confirmed via Fourier-transform infrared spectroscopy (FTIR; Bruker TENSOR 27) in the range of 400–4000 cm. The thickness of the coatings was measured from Optical microscope (OM) cross‑sectional images using ImageJ software (National Institutes of Health, version 1.54p, USA, https://imagej.net/ij)^33^.

The (AC and DC) electrochemical investigations were performed using potentiostat (an Autolab PGSTAT100). The measurements were carried out in a three-electrode electrochemical cell with a platinum counter electrode and a saturated calomel electrode as a reference in SBF (simulated body fluid) solution as the corrosive medium at room temperature. Before the experiments, the working electrode (coated sample) was immersed in the test solution for 30 min to establish a steady state open circuit potential. Electrochemical impedance spectroscopy measurements were carried out in the frequency range of 100 kHz to 10 mHz using an amplitude of 5 mV peak-to-peak ac signals at the open circuit potential (E_corr_). The potentiodynamic polarization measurements were performed in a range of E_corr_ ± 300 mV, and the potential was changed from cathode to anode values with a scan rate of 0.1 mV s. The equivalent electrical circuit was developed based on experimental data using the Nyquist plot, and the EIS results were analyzed using Zview software (Scribner Associates Inc., version 3.5c, https://www.scribner.com/products/zview)^34^.

## Results and discussion

### Powder characterizations

**Fig. 3:**
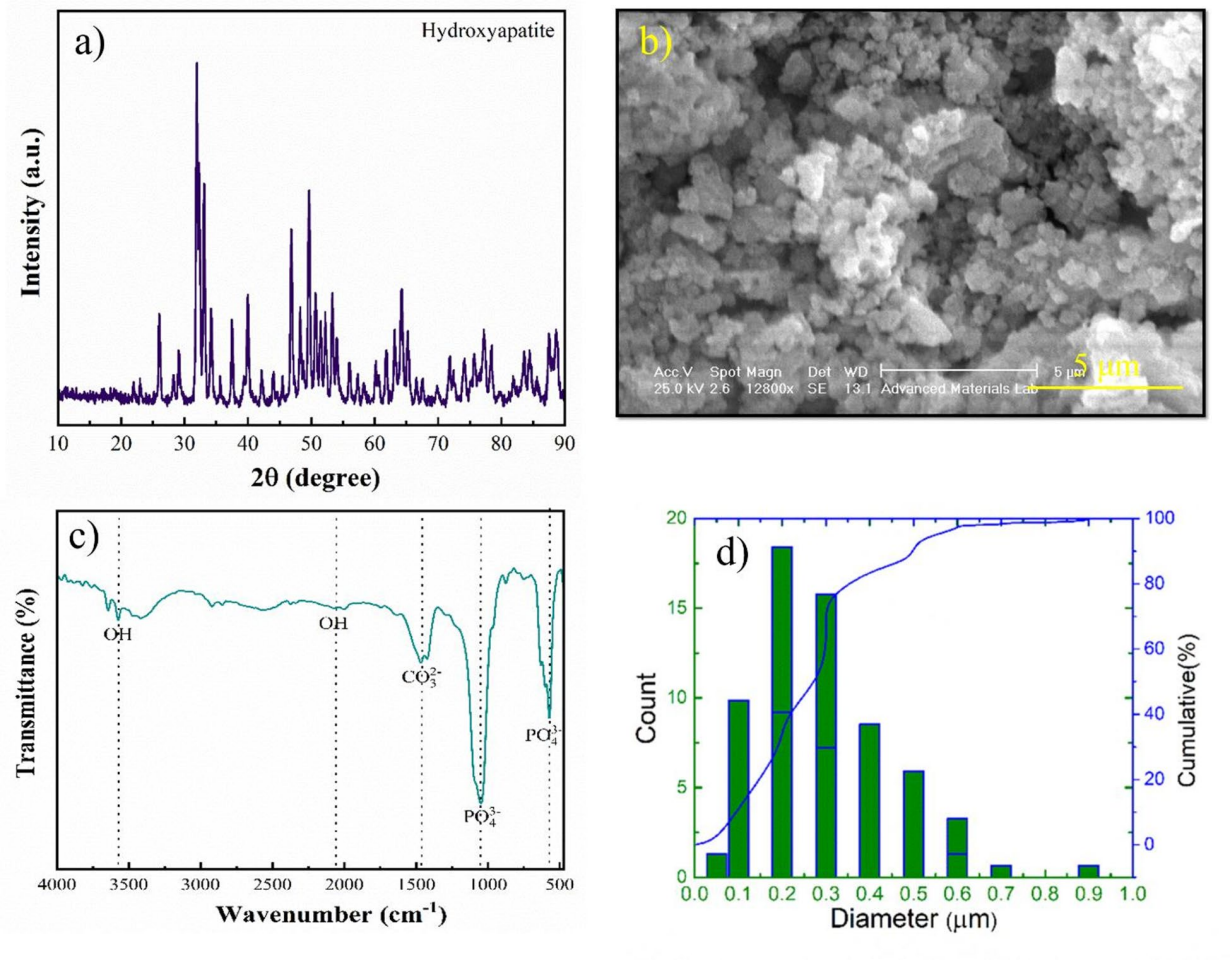
Characterization data of the HA nanopowders synthesized via the sol-gel method: (**a**) XRD pattern, (**b**) SEM micrograph, (**c**) FTIR spectrum, and (**d**) particle size distribution curve.

The XRD pattern of HA nanopowders synthesized by the sol-gel method is shown in Fig. 3a. All the diffraction pattern peaks using Bragg’s laws have been held as indices. The XRD patterns of the obtained powder showed only the sharp peaks of HA (JCPDS 96-901-0052). As can be seen, it is crystalline, and its main phase is hexagonal. Figure 3b shows the morphology of the HA nanopowder particles. Each particle consisted of a large number of nanosized spherical particles ranging from 50 nm to 200 nm. Fig. 3c shows the HA particle size distribution after calcination, obtained from SEM images. Analysis of the particle size distribution curve established the 10th, 50th, and 90th percentile sizes as 0.1 μm, 0.2 μm, and 0.3 μm, respectively. Infrared (IR) spectroscopy was used to identifying the chemical bands of the different phases as shown in Fig. 3d. FTIR analysis revealed several characteristic absorption features. Intense bands observed in the 900–1100 cm^− 1^ region are specifically attributed to the stretching vibrations of the $\:{\mathrm{P}\mathrm{O}}_{4}^{3-}$group within the HA lattice structure. Further vibrational modes of the phosphate moieties, namely the stretching and bending modes, were clearly identified at 612 cm^− 1^ and 580 cm^− 1^. The presence of the hydroxyl group was confirmed by the characteristic stretching mode peak detected at 3574 cm^− 135^. Furthermore, absorption peaks corresponding to carbonate groups $\:{\mathrm{C}\mathrm{O}}_{3}^{2-}$group located at 1422 cm^− 1^ and 1458 cm^− 1^, were registered, indicating their substitution for phosphate groups within the HA crystal structure. The absorption bands at 3448 cm^− 1^ and 1630 cm^-1^ could be attributed to adsorbed water and hydrocarbon impurity of powders, respectively^36^.

**Fig. 4:**
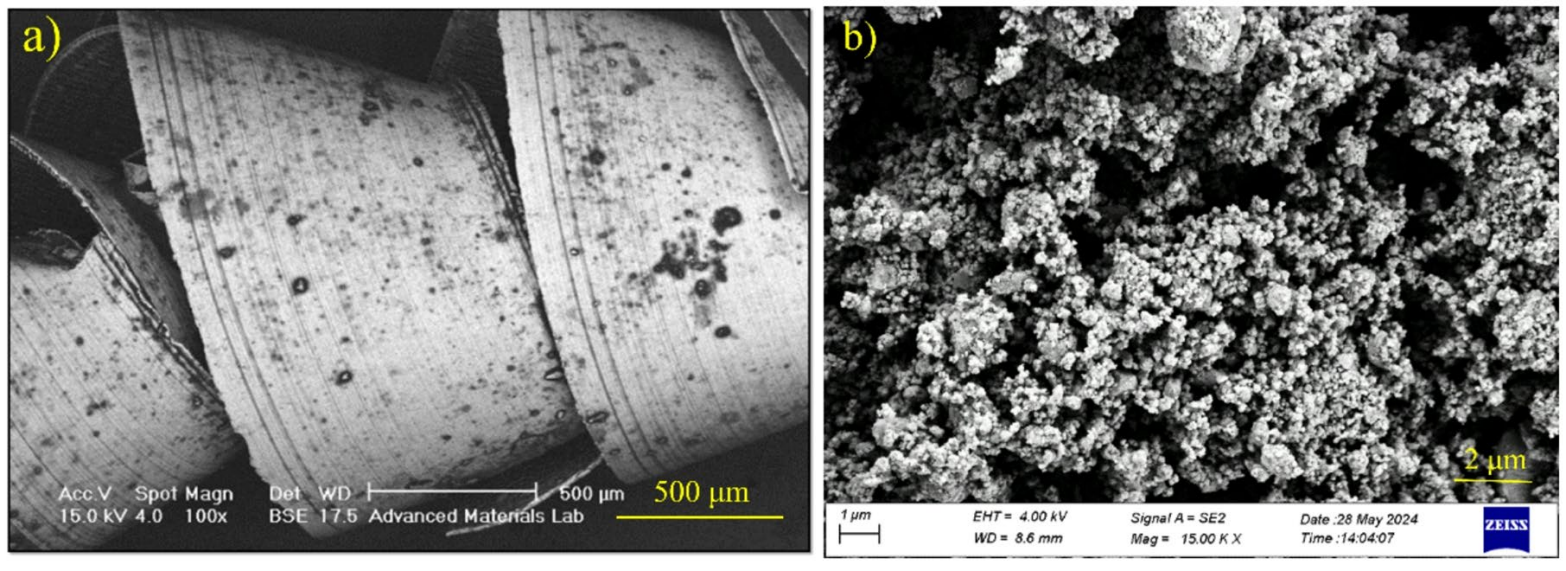
SEM micrographs comparing the surface morphology of (**a**) raw Ti6Al4V chips and (**b**) Ti6Al4V nanopowders produced through mechanical milling.

Also, Fig. 4 illustrates the initial titanium machining chips and the nanopowders produced through mechanical milling. The characteristics of the obtained powder have been thoroughly investigated in our previous work^12^. As observed, the milled powder exhibits good morphological uniformity with quasi-spherical particles and a significant reduction in particle size, providing a high specific surface area. According to previous studies^37–39^, mechanically milled powders generally demonstrate superior electrophoretic deposition (EPD) performance compared to unmilled or coarse-grained powders. This enhancement originates from multiple synergistic factors: (i) improved surface charge and suspension stability, (ii) Enhanced dispersibility and reduced agglomeration tendency, (iii) Optimized electrophoretic mobility. Consequently, suspensions formulated from ball-milled Ti-based oxide powders exhibit higher deposition rates, less particle clustering, and smoother, denser coatings compared to suspensions prepared from raw or coarser powders. This correlation between milling-induced surface activation and improved EPD behavior has been consistently reported in ceramic-metal composite systems such as TiO₂, HA, and Al₂O₃-based suspensions, confirming that mechanical activation substantially enhances the electrophoretic deposition capability of particles^38,40^.

### Phase and microstructural investigation of coating

#### Morphology and thickness of coating

**Fig. 5:**
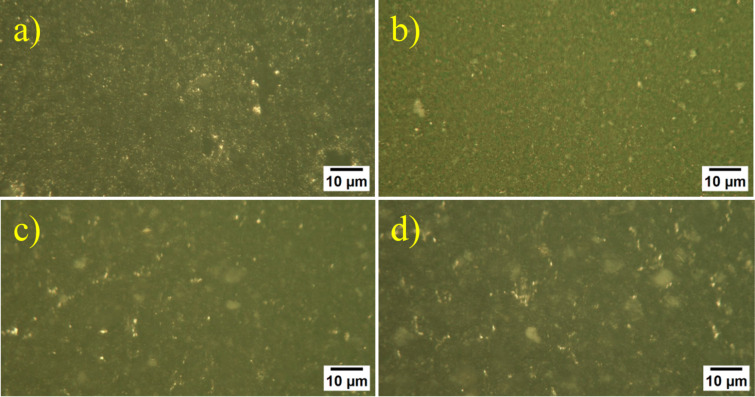
OM images of the HA-TiO_2_ coatings applied onto Ti6Al4V substrates with varying HA contents (10, 20, 30, and 40 wt%). All samples were processed using deposition parameters of 45 V for 90 s and subsequently sintered at 1100 ℃ for 4 h under vacuum.

**Fig. 6:**
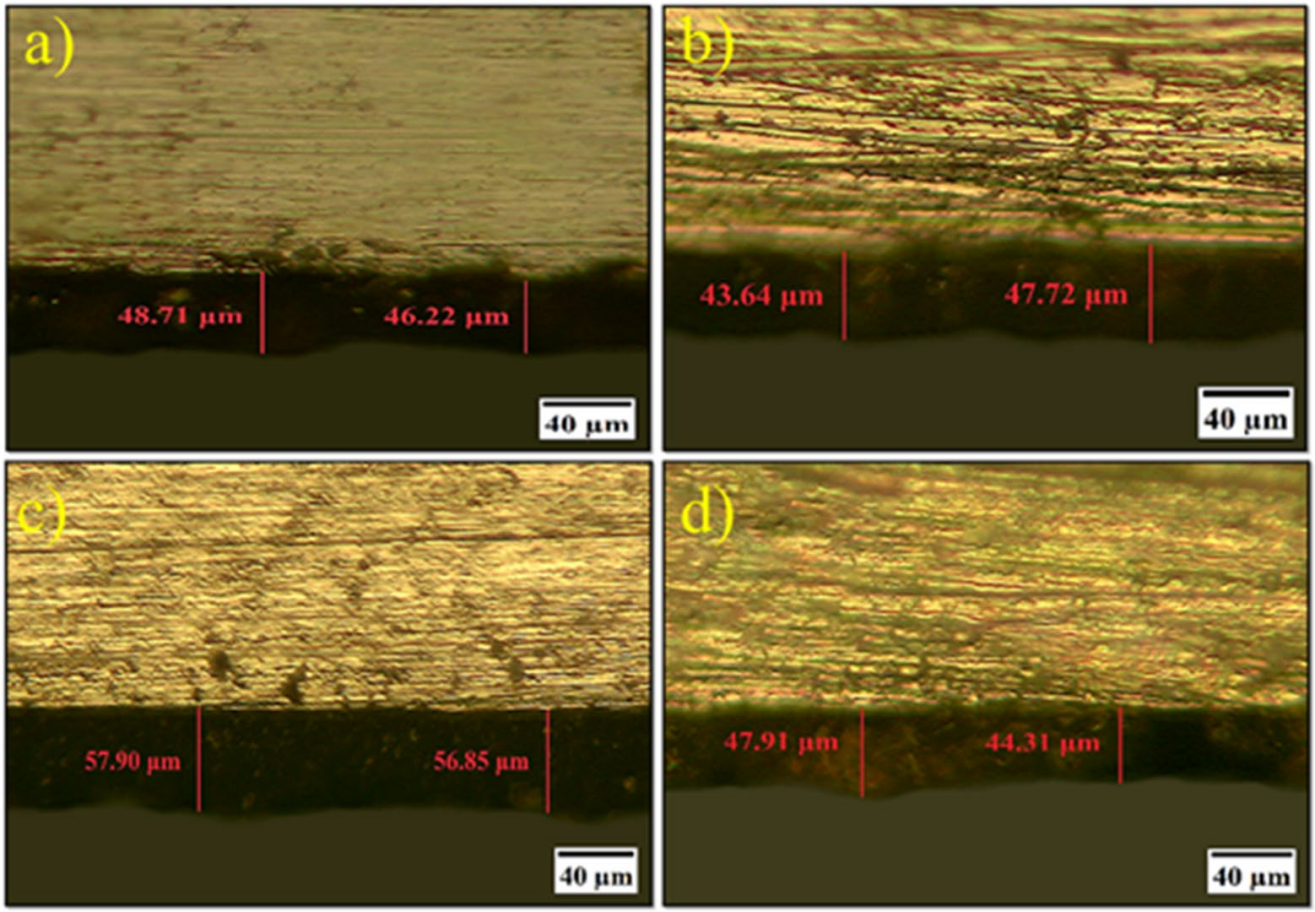
Cross-sectional OM images detailing the thickness and uniformity of the TiO_2_−HA coatings with different HA loadings: (**a**) 10 wt%, (**b**) 20 wt, (**c**) 30 wt%, and (**d**) 40 wt% HA. The EPD parameters were 45 V for 90 s.

Fig. 5 show the OM images of the HA-TiO_2_ coated samples with different HA values of 10, 20, 30, and 40 wt% deposited at a constant voltage of 45 V for 90 s, respectively. As shown in this figure, the 10 wt% HA coated sample was not a uniform coating. By increasing the HA to 30 wt% , the quality of the coating improved, but at higher amounts HA decreased again. The cross-section of these coatings is shown in Fig. 6. The thickness of the composite coatings produced from suspension containing 10, 20, 30, and 40 wt% HA was measured from the cross-sectional images using the ImageJ software. It was observed that increasing the HA concentration in the suspension led to an increase in coating thickness (from 43.64 to 57.9 μm); however, this thickness enhancement was accompanied by the formation of voids and porosity, which is undesirable for coating integrity. Also, in samples coated at higher concentration, which is the powders available for deposition are more, the deposition of coating material increased and led to higher thickness, from an average of 47 to 56 μm. Several articles have reported similar results, showing that the weight and thickness of the coating increase with increasing particle concentration in the suspension^39^.

**Fig. 7:**
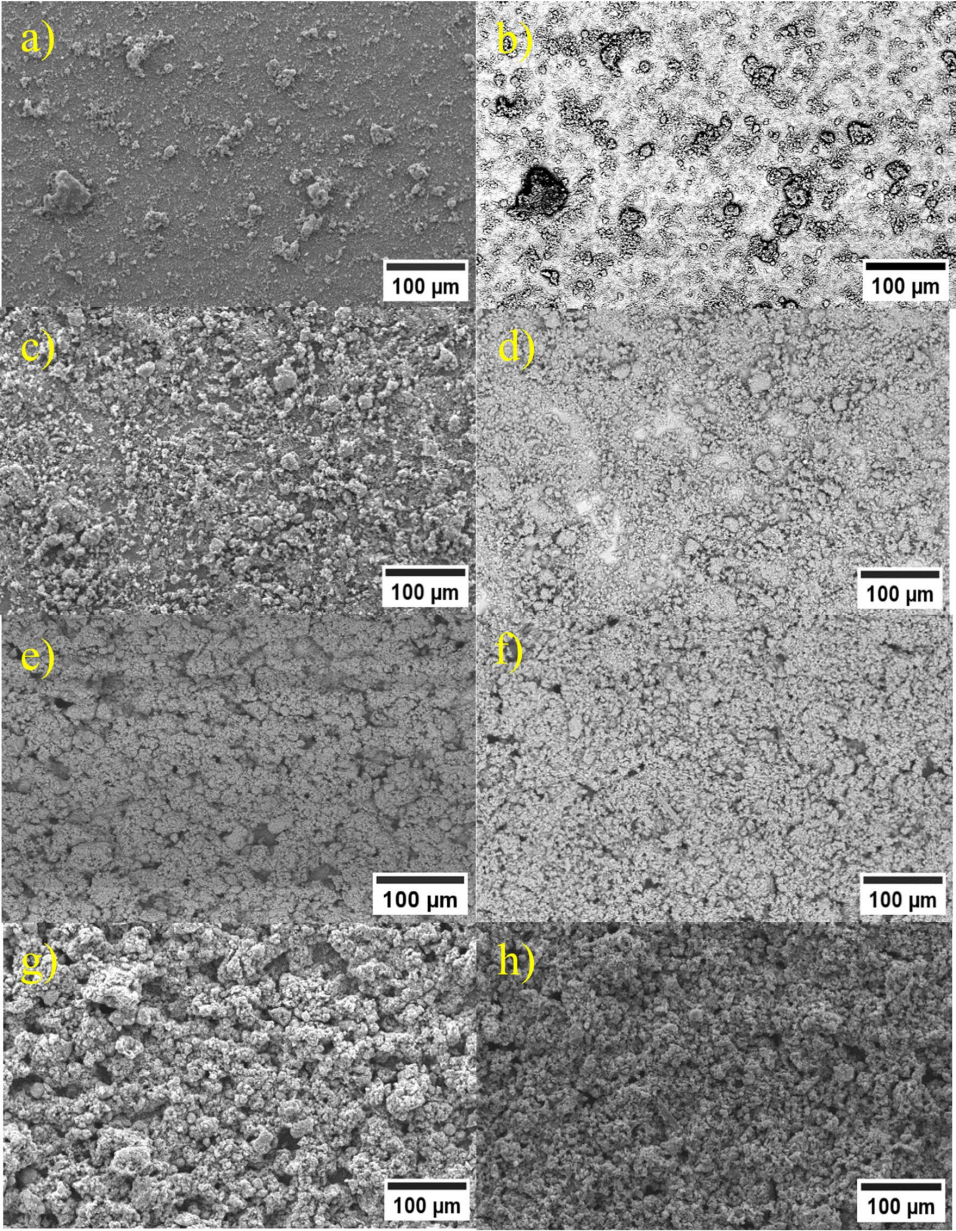
High-magnification SEM images illustrating the surface morphology of the HA-TiO_2_ ​ composite coatings deposited on Ti6Al4V substrates containing (**a**,** b**) 10 wt%, (**c**,** d**) 20 wt, (**e**,** f**) 30 wt%, and (**g**,** h**) 40 wt% HA. The coatings were sintered at 1100 ℃ for 4 h in a vacuum furnace.

For further investigation, SEM images with different magnifications were used, as shown in Fig. 7. Fig. 7 show the SEM images of the coated samples with different HA values of 10, 20, 30, and 40 wt% deposited at 45 V for 90 s (sintered samples). In the 10 wt% HA coated sample, the coating was not completely formed on some areas. Limited bright areas in the backscattered SEM images indicate that some limited and small areas have not been covered by EPD deposition. Uniform coating was observed in the sample coated with 30 wt% HA solution. All surfaces are well coated by electrophoretic deposition. By increasing the HA concentration to 40 wt% HA, although the coating formed well on the surface, the coating became more non-uniform. On the other hand, electrophoretic coating of one-component is somewhat different from two-component coating^38^. Our initial experimental results showed that although the suspension of TiO_2_ particles in ethanol is relatively stable, even with the addition of iodine as a dispersant, coating cannot be achieved. However, the quality of the coating is greatly improved by adding hydroxyapatite and increasing its concentration. At all hydroxyapatite concentrations, the zeta potential value is positive, indicating that the particles are positively charged. Increasing the amount of HA to 40 wt% has caused a decrease in zeta potential due to weakening of the electrical double layer. Similar results have been reported in the reduction of the zeta potential of particles due to the addition of high amounts of additives^41,42^.

During electrophoretic deposition, the cooperative migration of HA and TiO₂ particles is governed by their compatible surface charge characteristics and comparable electrophoretic mobility, which enable simultaneous movement toward the cathode. Furthermore, hetero-agglomeration between HA and TiO₂ particles through electrostatic interactions and surface adsorption leads to the formation of mixed agglomerates that migrate as composite entities, thereby promoting homogeneous co-deposition and enhanced packing efficiency.

Each particle type, like HA and TiO_2_, has a surface charge (governed by the zeta potential), a point of zero charge (PZC) or isoelectric point (IEP), and a surface chemistry (hydroxyl groups, adsorbed ions, etc.) which determines whether particles repel (stable) or aggregate (unstable) in the suspension^43,44^. Main surface groups for HA is –OH, Ca²⁺, PO₄³⁻ groups and typical IEP / PZC is ~ 6–7. Around pH ~ 6, both HA and TiO₂ have small or opposite zeta potentials; making easier co-deposition^45^. Therefore, HA and the TiO_2_ have compatible surface potentials, they migrate and deposit together uniformly under an electric field. This fact is further improved by increasing the HA concentration in suspension. In fact, more particles are provided with the opportunity to migrate and form a dense, uniform coating. At 40HA, Ca²⁺ from HA can adsorb onto negatively charged ceramic surfaces (TiO₂–O⁻), forming Ca²⁺ bridges and particle clustering occurs.

#### XRD analysis of coating

**Fig. 8:**
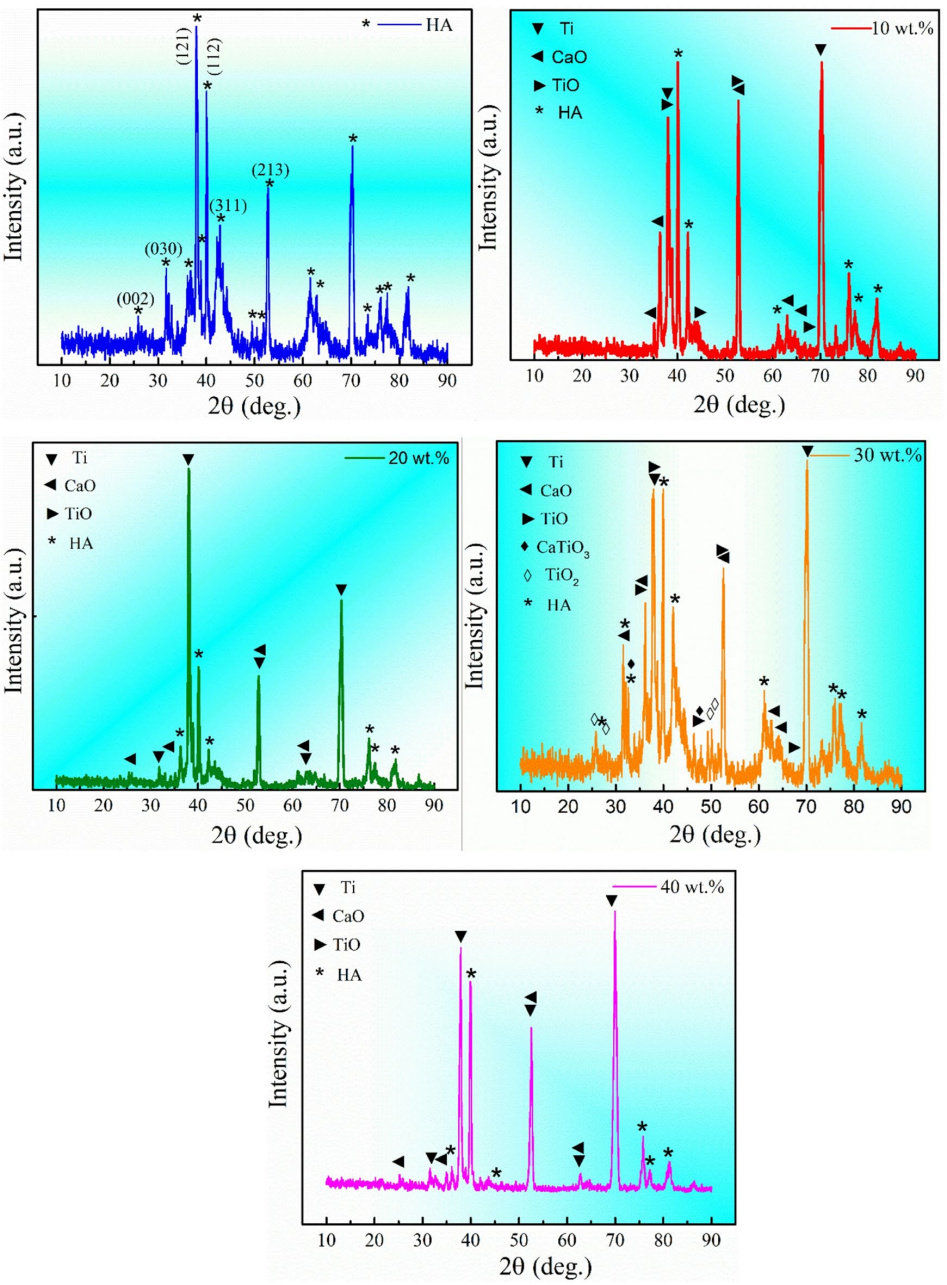
XRD patterns of the HA–TiO_2_ ​composite coatings on the Ti6Al4V substrate after sintering at 1100 ℃. The patterns correspond to coatings containing: ( 0 wt%, 10 wt%, 20 wt%, 30 wt% and (e) 40 wt% HA)

The XRD patterns of the HA–TiO_2_ coatings, deposited on the Ti6Al4V substrate and sintered at 1100 ℃, are presented in Fig. 8 for various HA concentrations (0 to 40 wt% HA). The phase identification was performed by matching the characteristic diffraction peaks with standard reference patterns, and the main peaks of each phase are explicitly marked in Fig. 8 to avoid ambiguity. Upon high-temperature processing, the resulting phases identified in the coatings, in addition to the primary HA and substrate (Ti and Ti6Al4V alloy peaks), included TiO_2_​, TiO, CaO, and calcium titanate (CaTiO_3_​).

In XRD pattern, titanium oxide peaks (36.21 ^o^ (111), 42.06 ^o^ (200), 60.99 ^o^ (220), 73.04 ^o^ (311), and 76.86 ^o^ (222)) emerged (JCPDS Card No. 01-086-2352). The weak peaks could be assigned to substoichiometric TiOₓ phases, overlapping with TiO₂ reflections. Additionally, peaks at 35.25 ^o^ (010), 38.68 ^o^ (002), 40.38 ^o^ (011), 53.32 ^o^ (012), 63.27 ^o^ (110), 71.15 ^o^ (013), and 77.79 ^o^ (021) indicated the presence of titanium (Ti) related peak (JCPDS Card No. 96-151-2548). All diffraction peaks align with the hexagonal HA phase (JCPDS 96-901-0052), indicating high phase purity and crystallinity.

During the post-deposition sintering process, phase transformation and interfacial bonding between the HA-based coating and the Ti substrate are governed by solid-state diffusion and thermodynamically driven interfacial reactions. At elevated temperatures, mutual diffusion of Ca, Ti, and O species across the coating–substrate interface becomes favorable, leading to the formation of reaction products such as CaTiO₃, as evidenced by the XRD results.

The formation of this interfacial phase reduces the chemical potential mismatch between the ceramic coating and the metallic substrate, thereby lowering interfacial energy and improving adhesion strength. Simultaneously, TiO₂ particles embedded within the HA matrix can act as diffusion facilitators and nucleation sites, promoting localized reactions and enhancing the mechanical interlocking at the interface.

It is concluded that a solid-state reaction occurs between hydroxyapatite (HA) and titanium oxide (TiO_2_) particles during the high-temperature sintering process, leading to the formation of calcium titanate (CaTiO_3_). Furthermore, the presence of the TiO_2_ ​phase is attributed to the partial re-oxidation of substoichiometric TiOₓ species during the subsequent cooling stage. Critically, the transformation of Ti (or its oxides) to TiO_2_​ results in a volumetric expansion that effectively counteracts the thermal densification shrinkage inherent to the heat treatment, thereby reducing in the overall porosity of the resultant film^46^. During sintering, local oxygen activity at the coating–substrate interface is reduced due to limited oxygen diffusion through the HA layer, which may promote the transient formation of substoichiometric titanium oxides (TiOₓ, x < 2). Upon cooling and exposure to air, these phases tend to re-oxidize toward thermodynamically stable TiO₂. Therefore, the detected oxide phases should be considered as part of a dynamic oxidation–re-oxidation process rather than distinct, static compounds. The results revealed that the milling process induced shifts in the peak positions. The reason for the observation of the CaO phase could be due to the partial decomposition of hydroxyapatite during sintering, according to the following reaction^47^:

$${\rm C{a_{10}}{\left( {P{O_4}} \right)_6}{\left( {OH} \right)_2}+Ti{O_2}=3C{a_3}{\left( {P{O_4}} \right)_2}+CaTi{O_3}+{H_2}O}$$

Also, HA phase preferentially react with anatase phase rather than rutile when TiO_2_-HA composite was sintered at T_sin_> 900℃ which leads to formation of CaTiO_3_^48^.

  

$${\rm C{a_{10}}{\left( {P{O_4}} \right)_6}{\left( {OH} \right)_2}=3\beta-C{a_3}{\left( {P{O_4}} \right)_2}+CaO+{H_2}O}$$

Formation of low contents of β-TCP is advantageous as it allows ionic substitutions and.

the bioactivity of the material may be enhanced^49^.

### Corrosion resistance of coating in SBF’s solution

#### Electrochemical polarization

**Fig. 9:**
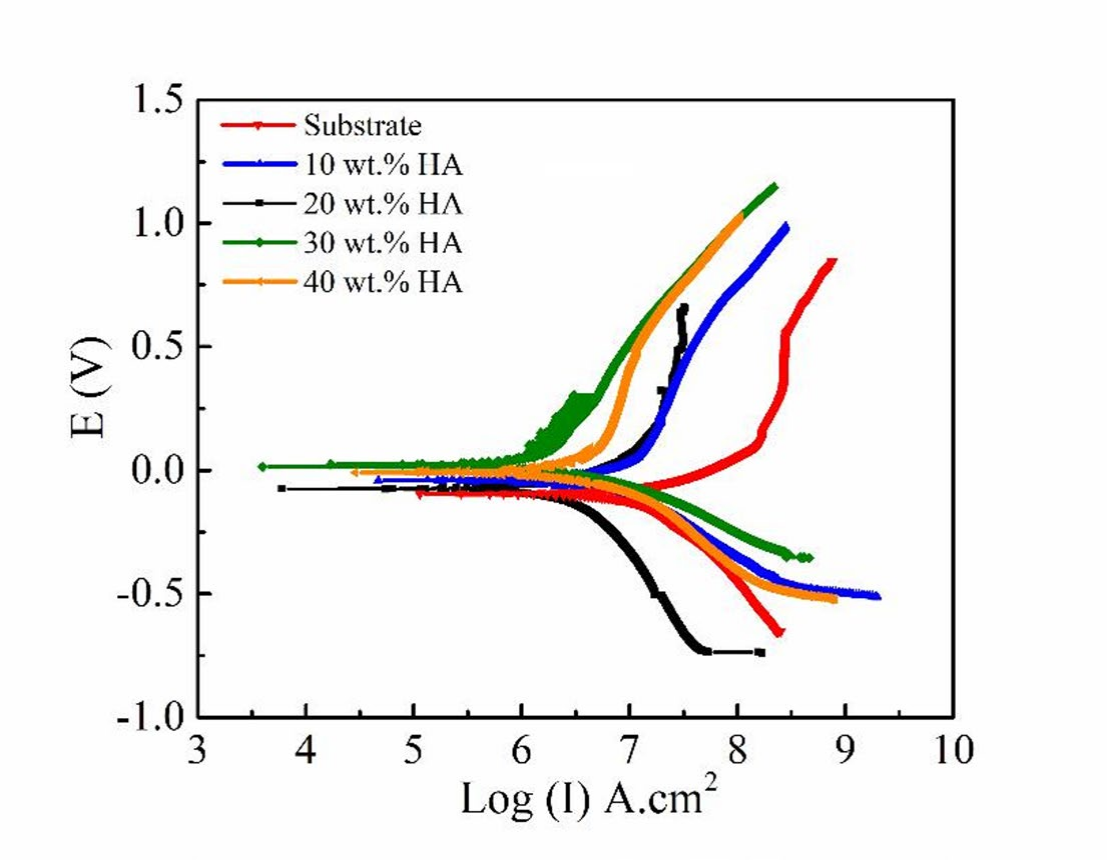
Potentiodynamic polarization Tafel curves for the uncoated Ti6Al4V alloy and the coated alloys (a) 0 wt%, (b) 10 wt%, (c) 20 wt%, (d) 30 wt% and (e) 40 wt% HA tested in simulated body fluid (SBF).

The potentiodynamic polarization response of the bare Ti6Al4V and the HA-TiO_2_ coatings (0 to 40 wt% HA) in SBF is graphically presented in Fig. 9. The corresponding electrochemical metrics (E_corr_​ and i_corr_), obtained via Tafel extrapolation, are systematically tabulated in Table 1. Comparison of polarization curves of different coatings and data from Table 1 shows that the corrosion potential of all composite coatings is more positive than the corrosion potential of the substrate. A clear improvement in corrosion resistance across all coated configurations was observed compared to the uncoated substrate, characterized by higher E_corr ​_ values and lower i_corr_ ​ values, which is indicative of the barrier properties imparted by the oxide/HA layer against electrolyte ingress. Based on these findings, the coating formulated with 30 wt.%HA, showed the most stable electrochemical response, resulting in the lowest measured corrosion rate (0.006 mm/year).

This behavior can be primarily attributed to differences in coating microstructure, as increased porosity enlarges the effective surface area exposed to the electrolyte and accelerates corrosion through preferential pore-mediated pathways. Obviously, corrosion through pores can lead to faster contact of the solution with the substrate and its severe corrosion. Also, comparison among the 20 wt% HA and 30 wt% HA coated samples shows that, corrosion potential of the 30 wt% HA (0.1231) is higher. This can be attributed to the high thermodynamic stability of the TiO_2_ phase formed in this coating after sintering. Therefore, the presence of TiO_2_ in the XRD pattern of 30HA coating increases the corrosion potential. Conversely, the sample incorporating the highest HA concentration (40 wt%) exhibited a measurable decline in corrosion resistance. This performance degradation correlates directly with microstructural observations **(**as depicted in the SEM micrographs, Fig. 7**)**, which indicate the formation of an increased density of isolated pores and a significant loss of continuity within the deposited layer. Consequently, although the mass fraction of HA was maximized, the insufficient interfacial bonding and poor particle interlock among the constituents resulted in poor consolidation of the coating, leading to a highly porous structure vulnerable to electrolyte penetration. Based on previous studies, it can be concluded that the corrosion current density of coated titanium implant alloys (Ti6Al4V) depends on the corrosion condition^50^.

**Table 1:** Summary of the electrochemical kinetic parameters derived from Tafel polarization measurements for the uncoated Ti6Al4V and the HA-TiO_2_ ​ coated samples HA. (a) 0 wt%, (b) 10 wt%, (c) 20 wt%, (d) 30 wt% and (e) 40 wt% HA tested in SBF.

		R_p_ (Ω.cm^2^) × 10^4^	E_corr_ V	i_corr_ µA.cm^− 2^	b_a_ (mV per decade)	b_c_ (mV per decade)	Corrosion rate mm/y
SAMPLES	sub	0.1395	-0.503	0.0001264	2.81	0.47	0.41
	10 wt% HA	1.152	-0.038	7.725	0.76	0.28	0.0252
	20 wt% HA	2.174	-0.297	8.97	1.5	0.64	0.0293
	30 wt% HA	3.551	0.1231	1.89	0.53	0.21	0.006
	40 wt% HA	2.713	0.1068	4.028	0.77	0.37	0.013

#### Electrochemical impedance spectroscopy (EIS)

**Fig. 10:**
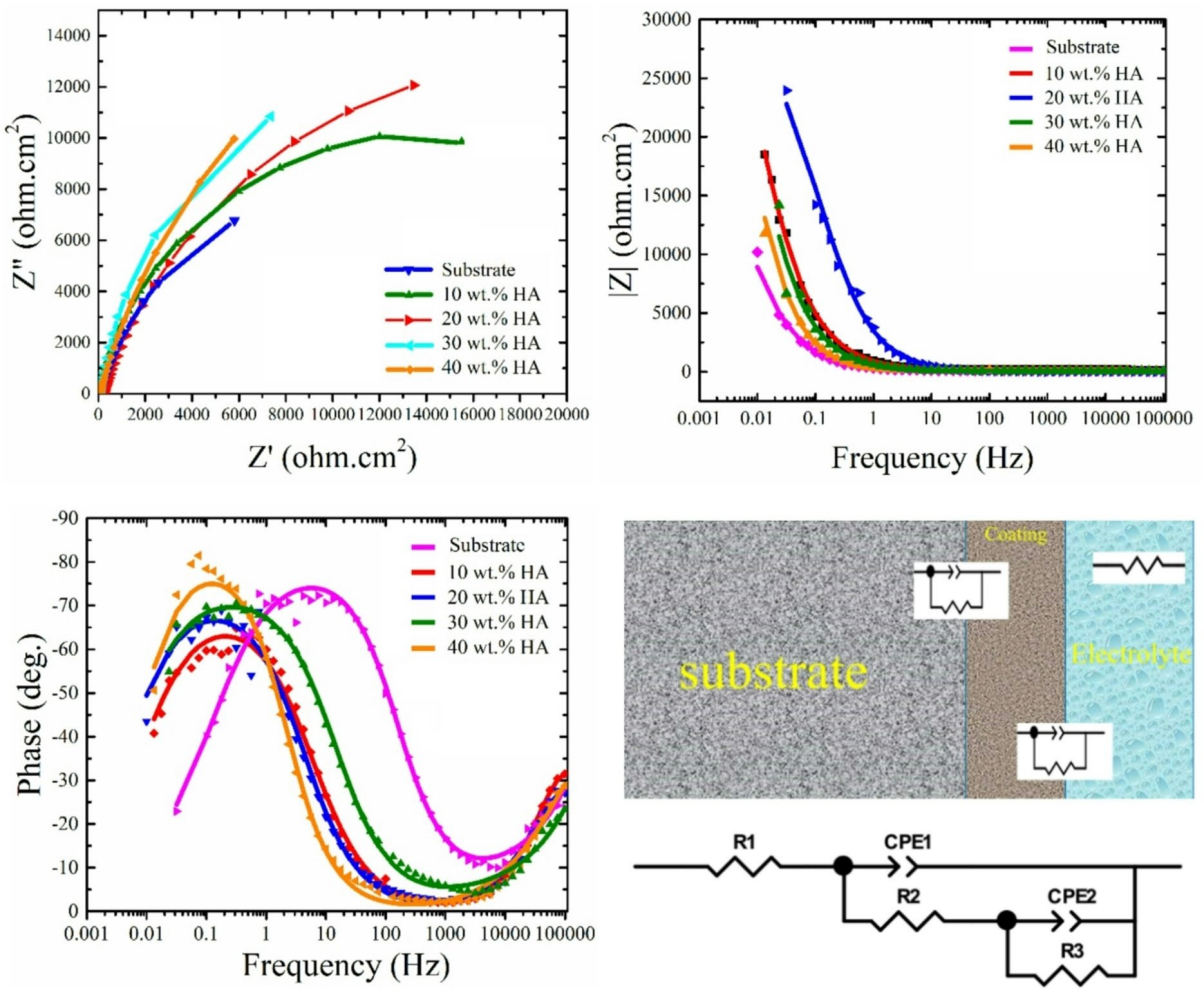
Electrochemical Impedance Spectroscopy (EIS) results in SBF: (**a**) Nyquist plots, (**b**) Bode magnitude plots, and (**c**) Bode phase plots, showing the experimental data and the fit obtained from the equivalent electric circuit model for the uncoated Ti6Al4V and coated samples ((a) 0 wt%, (b) 10 wt%, (c) 20 wt%, (d) 30 wt% and (e) 40 wt% HA). (**d**) Schematic illustration of the equivalent electric circuit model used for fitting the impedance response.

The electrochemical impedance spectroscopy (EIS) measurements, including the Nyquist (Fig. 10a), Bode amplitude and phase (Fig. 10b and c), and associated theoretical model fitting plots, were conducted on all samples immersed in simulated body fluid (SBF). In the Nyquist representation (Fig. 10a), all coated samples (0 to 40 wt% HA) exhibited a characteristic two-time-constant response: a small capacitance loop at high frequencies, succeeded by a larger diameter semicircle in the low-frequency domain. The equivalent electrical circuits (EEC) employed for fitting the impedance spectra of the bare and coated substrates are illustrated in Fig. 10d. The equivalent electrical circuit employed to fit the EIS data consists of two constant phase elements (CPEs), each representing a distinct electrochemical process occurring at different length scales. The high-frequency CPE is associated with the response of the coating layer, reflecting its dielectric behavior, surface heterogeneity, and non-ideal capacitive characteristics arising from porosity and roughness. In contrast, the low-frequency CPE corresponds to the electrochemical processes at the coating/substrate interface, including charge transfer reactions and double-layer capacitance. The use of two CPE components is therefore necessary to decouple the electrochemical response of the porous HA-based coating from the interfacial charge transfer behavior, allowing for a more accurate representation of the impedance spectra and improved fitting quality^51^.

Crucially, a comparative analysis of the Nyquist plots demonstrated a significant increase in the diameter of the low-frequency semicircle (representing polarization resistance, Rp ​) as the HA content increased from 0 to 30 wt%. This increase is indicative of enhanced corrosion resistance due to the formation of a more effective barrier layer that restricts the penetration of corrosive species to the underlying metallic substrate. The 30 wt% HA coating yielded the maximum polarization resistance; notably, the 40 wt% HA sample showed a slight reduction in this radius, a finding consistent with prior microstructural and polarization analysis. From a physicochemical and microstructural perspective, the superior corrosion performance observed at 30 wt% HA arises from an optimal balance between particle concentration, coating compactness, and structural integrity. At this composition, effective particle packing minimizes interconnected porosity and restricts ionic transport, whereas excessive HA contents promote agglomeration and microstructural heterogeneity that compromise barrier performance.

In the Bode phase plot (Fig. 10c), two distinct peaks were resolved for all samples, corresponding to the two time constants observed in the Nyquist plot, situated approximately between 10,000 and 100,000 Hz and 0.1 to10 Hz. The phase angle magnitude became progressively more negative with increasing HA content up to 30 wt%. Furthermore, the characteristic phase peak of the bare substrate shifted toward lower frequencies upon coating. The 30 and 40 wt%HA composition exhibited the highest maximum phase angles at low frequencies, a feature often correlated with superior dielectric behavior and reduced charge transfer, further confirming their superior barrier performance^51^. These results were corroborated by the Bode impedance plot (Fig. 10b), where the highest overall impedance magnitude was recorded for the 30 and 40 wt% HA systems. The estimation of coating capacitance, an intrinsic material characteristic, was achieved by fitting the experimental data to the appropriate EEC models (Fig. 10d; Table 2) using ZView software, which provided an excellent fit across all measured spectra. The low χ² values and minimal residuals confirm the good agreement between the experimental data and the fitted equivalent circuit.

In the selected equivalent circuit, instead of using an ideal double-layer capacitance, another circuit element called a constant-phase element (CPE) is used, which is a non-ideal capacitor. The impedance of CPE is defined by Eq. (5), as follows^41^:

5 $${Z_{CPE}}=\frac{1}{{{{\left( {j\omega } \right)}^n}Q}}~~$$

where j = √-1, *ω* = 2*πf*, n and Q are frequency-independent and depend on temperature. The exponent *n* of the CPE, 0 ≤ *n* ≤ 1, is related to the surface roughness. If *n* = 1, the CPE is an ideal capacitor, whereas if n is zero, the CPE behaves similar to an ideal resistor. The data in Table 2 clearly show that R_s_ (the resistance of the electrolyte solution) values in the various wt% HA solutions are similar to each other (in the range of 19–55 Ω.cm^2^). However, a higher R_P_ value indicates a higher corrosion resistance of the 30 wt% HA, indicating that metal ions are released into the SBF solutions at lower rates. Analysis of the equivalent circuit parameters presented in Table 2 reveals that the charge transfer resistance R_ct_​, denoted as R_3_​ in the model) reached its maximum value for the coating incorporating 30 wt% HA. This maximal R_ct_ directly quantifies the superior resistance to electrochemical reactions provided by this specific formulation. The overall EIS findings are in robust agreement with the conclusions drawn from the potentiodynamic polarization studies (Tafel analysis). Any minor quantitative discrepancy between the two techniques can be attributed to inherent methodological differences, specifically the constraints imposed by the EEC fitting procedure in EIS and the reliance on Tafel extrapolation slope calculations for the polarization curves.

**Table 2:** Electrochemical parameters calculated by modeling the EIS data for the TiO_2_−HA coatings as a function of HA content (χ² is included to demonstrate the quality and reliability of the EIS fitting).

*R*_1_ (Ω.cm^2^)	Q (µF.cm^− 2^)	*R*_2_ (Ω.cm^2^)	*n*	Q (µF.cm^− 2^)	*R*_3_(MΩ.cm^2^)	*n*	χ²	
sub	19	5.8E-7	62	0.7	0.0008526	21,404	0.8	0.006
10 wt% HA	3.72	0.000001	135	0.6	0.0006308	29,077	0.7	0.009
20 wt% HA	55	4.8422E-8	155	0.7	0.00028049	39,755	0.7	0.008
30 wt% HA	40	0.00000006	2884	0.3	0.0003735	46,823	0.8	0.007
40 wt% HA	42	2.49E-5	199	0.2	0.0000489	43,604	0.9	0.006

**Fig. 11:**
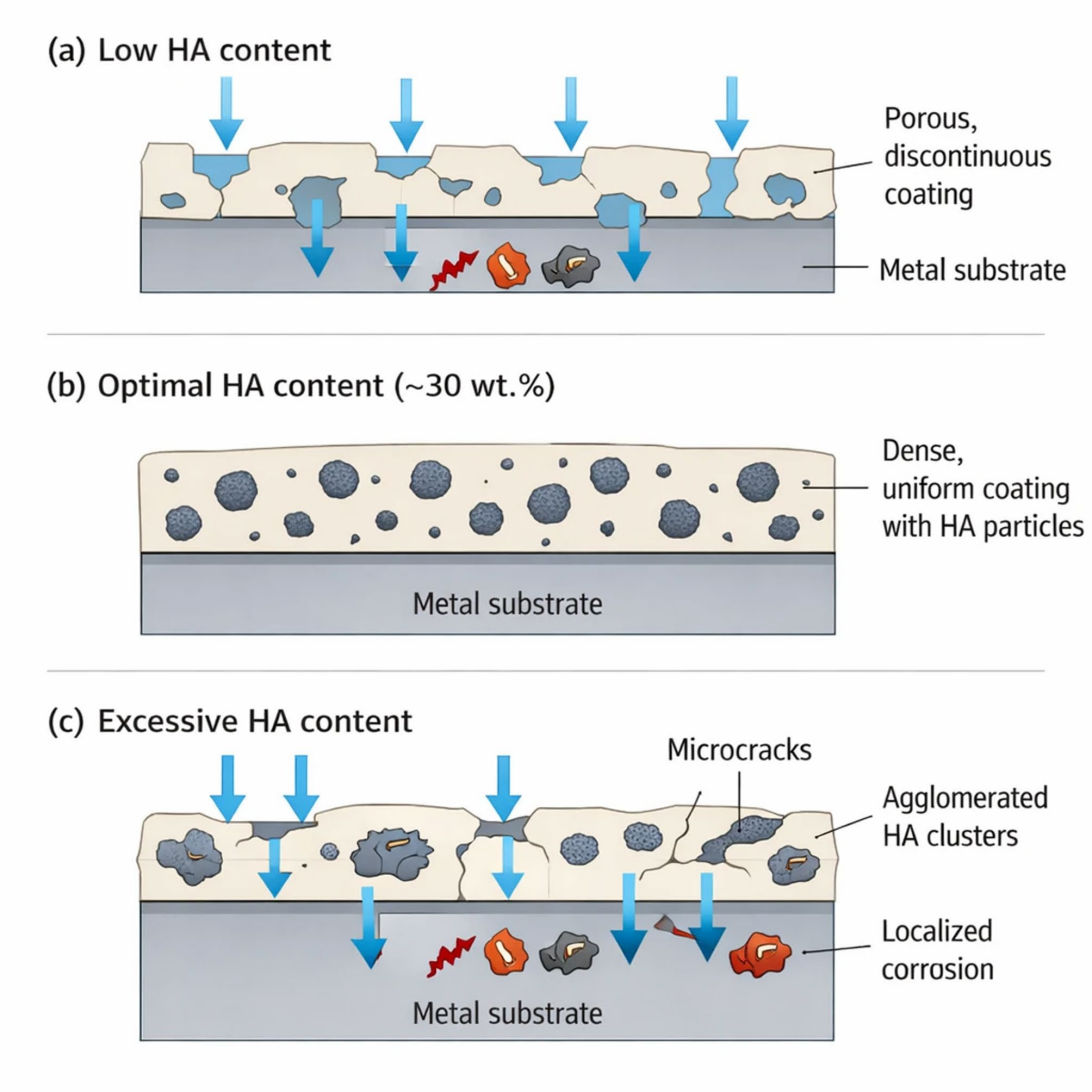
Schematic illustration of the influence of HA content on coating microstructure and the corresponding corrosion protection mechanism: (**a**) low HA content yields a porous/discontinuous coating enabling electrolyte penetration and interfacial corrosion; (**b**) an optimal HA content (~ 30 wt%) forms a dense and compact barrier layer that effectively blocks ionic transport; (**c**) excessive HA content promotes particle agglomeration and localized defects (e.g., microcracks), facilitating localized electrolyte access and corrosion.

The observed variation in corrosion behavior with changing HA content can be directly correlated with the evolution of the coating microstructure. At low HA contents, the coating remains relatively porous and discontinuous, providing limited barrier protection and allowing electrolyte penetration through interconnected defects. As the HA content increases, improved particle packing and enhanced coating compactness effectively block ionic transport pathways, leading to increased charge transfer resistance and reduced corrosion current density. To further clarify this structure–property–corrosion relationship, a schematic illustration of the corrosion protection mechanisms as a function of HA content is presented in Fig. 11. However, at excessively high HA contents, particle agglomeration and reduced inter-particle cohesion result in microstructural heterogeneities and localized defects, which facilitate electrolyte access to the substrate. This structural degradation compromises the protective function of the coating, despite the higher HA fraction. Consequently, an optimal HA content (e.g., 30 wt%) achieves a balance between coating densification and structural integrity, thereby providing the most effective corrosion protection.

## Conclusion

In this study, a novel dual synthesis approach was successfully employed to produce a surface-engineered powder with enhanced corrosion resistance. The optimized coating process led to the formation of a uniform and adherent protective layer, effectively minimizing degradation under corrosive environments. Electrochemical evaluations confirmed that the coated specimens exhibited significantly improved stability and lower corrosion rates compared to the uncoated ones. Overall, the presented methodology demonstrates a promising route toward designing advanced surface materials for harsh-service applications, offering both structural integrity and long-term durability. An optimal HA content (~ 30 wt%) was identified, providing a dense and compact coating with superior corrosion resistance, as supported by electrochemical analyses and the proposed mechanistic model.

## Data Availability

Data will be made available on request by sending an email to corresponding author.
